# An Unusual Presentation of a Left Extra-acetabular Trochanteric Bursa Abscess

**DOI:** 10.7759/cureus.103733

**Published:** 2026-02-16

**Authors:** Mackenzie J Ewart, Shanzay Mohammad, Daniel Erickson, Brett Platis, Justinne Certeza, Jake Powell, Fatima Khan, Barbara Karnkowska, Ivan Marrufo, Cara L Fisher

**Affiliations:** 1 Department of Surgery, Texas Health Harris Methodist Hospital, Fort Worth, USA; 2 Department of Paediatrics, Methodist Children’s Hospital, San Antonio, USA; 3 Department of Emergency Medicine, Geisinger Medical Center, Danville, USA; 4 Department of Family Medicine, University of California Los Angeles Medical Center, Los Angeles, USA; 5 Department of Family Medicine, University of Pittsburgh Medical Center, Pittsburgh, USA; 6 Department of Orthopaedics, John Peter Smith (JPS) Health Network, Fort Worth, USA; 7 Department of Radiology, University of Texas Southwestern Medical Center, Dallas, USA; 8 Department of Internal Medicine, University of Texas Medical Branch, Galveston, USA; 9 College of Osteopathic Medicine, University of North Texas Health Science Center, Texas College of Osteopathic Medicine, Fort Worth, USA; 10 Oral Biology, Creighton University, Omaha, USA

**Keywords:** cadaveric study, histopathology, lateral trochanteric pain, synovial osteochondromatosis, trochanteric bursitis

## Abstract

Trochanteric bursitis is a common cause of lateral hip pain, particularly among elderly and postmenopausal females, but abscess formation within the trochanteric bursa is exceedingly rare. We report an unusual extra-acetabular trochanteric abscess identified during cadaveric dissection of an elderly female, characterized by irregular fibrotic and proteinaceous tissue with multiple inclusion bodies but no evidence of malignancy or granulomatous inflammation. Although limited by an absent clinical history, the gross and histopathologic findings suggest a chronic inflammatory or degenerative process consistent with synovial osteochondromatosis of the trochanteric bursa.

## Introduction

The extra-acetabular region of the hip encompasses the peri-trochanteric soft tissues surrounding the greater trochanter, including the trochanteric bursae, gluteus medius and gluteus minimus tendons, and adjacent fascial structures, which lie outside the true synovial hip joint capsule. Trochanteric bursitis represents sterile inflammation of a peri-trochanteric bursa [1]. Trochanteric bursitis is a common cause of lateral hip pain, particularly in elderly and postmenopausal women, and is typically associated with repetitive stress, acute injury and trauma, poor biomechanics, particularly with tendon/iliotibial band pathology, muscle weakness such as back pain, obesity, osteoarthritis, and compressive irritation [2,3]. In contrast, abscess formation or mass-like pathology involving the trochanteric acetabular bursa is rare and may mimic neoplastic, infectious, or inflammatory processes [4,5]. Abscesses usually have localized purulent infection within soft tissue, arising from the intra-articular synovial lining of the hip joint capsule [6]. In this study, we utilized histopathological tissue analysis as well as the patient’s past medical history to identify the clinical condition underlying the abnormal tissue and stones.

## Case presentation

**Figure 1:**
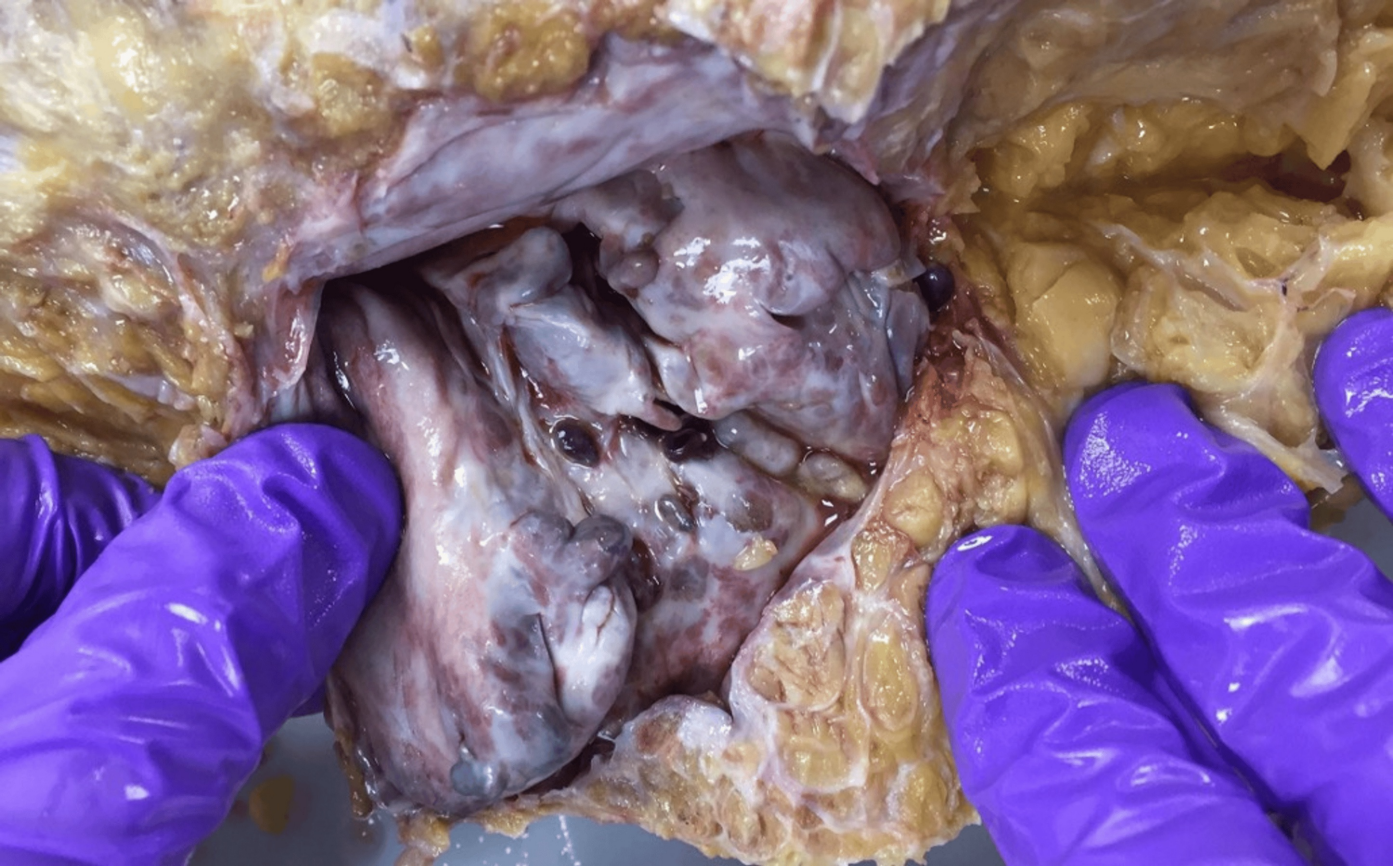
The bursa over the donor’s left greater trochanteric region following drainage of fluid.

Our research subject was a 79-year-old female with a history of hypertension, heart murmur, left bundle branch block, and mental confusion. Prior surgeries included a hemorrhoidectomy, hysterectomy, lumpectomy, and a mastectomy. Following exposure of the left hip and proximal femur, an abnormal fluid-filled cavity was identified adjacent to the trochanteric bursa near the ischium and ilium (Figure 1).

**Figure 2:**
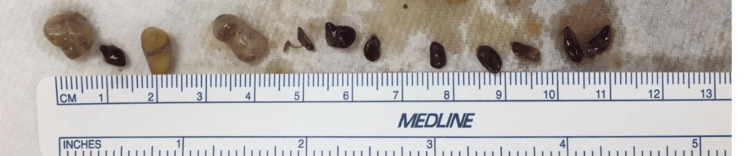
Loose bodies found within the site of the abscess.

The area was carefully dissected to preserve tissue architecture. Nonpathological surrounding tissue was removed, and the anomalous contents, including loose bodies and nodules, were photographed (Figure 2) and collected for analysis.

Four 2 cm² tissue samples were excised from the inner bursal surface of the trochanteric abscess and were immediately fixed in 50 mL of formalin for 48 hours. Specimens were processed at the University of North Texas Health Science Center (UNTHSC) histology laboratory. Serial sections were prepared using standard paraffin embedding and stained with hematoxylin and eosin (H&E).

Microscopic examination was performed at multiple magnifications (4× to 40×). Photomicrographs were captured using an Olympus digital pathology system. Slides demonstrating prominent pathological features, including inclusion bodies, fibrosis, and vascularity, were selected for further description.

**Figure 3:**
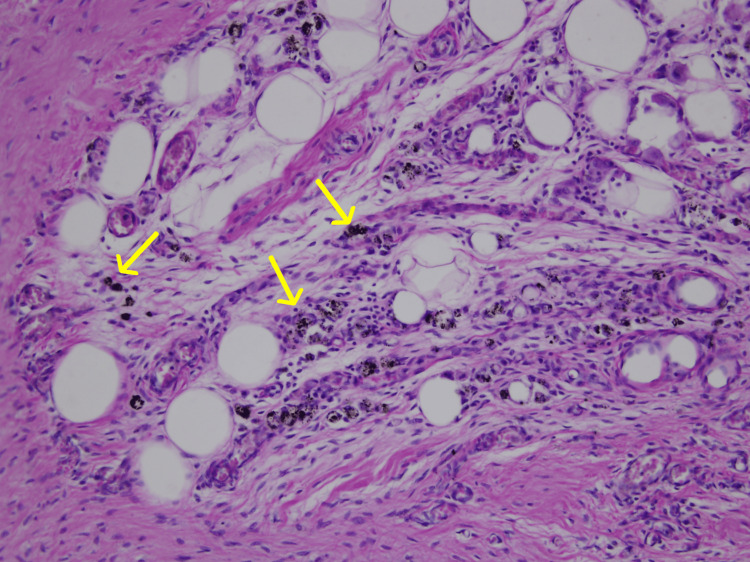
Proteinaceous inclusion bodies (yellow arrows) (40×, Sample 19-45).

Gross inspection revealed irregular yellow-brown soft-tissue fragments, multiple small nodules, and a cavity consistent with a chronic bursal abscess. Histological examination demonstrated dense fibrotic stroma interspersed with adipose tissue, small-caliber vessels, and scattered proteinaceous inclusion bodies. In Sample 19-45, numerous darkly pigmented, round-to-oval inclusions were observed (Figure 3).

**Figure 4:**
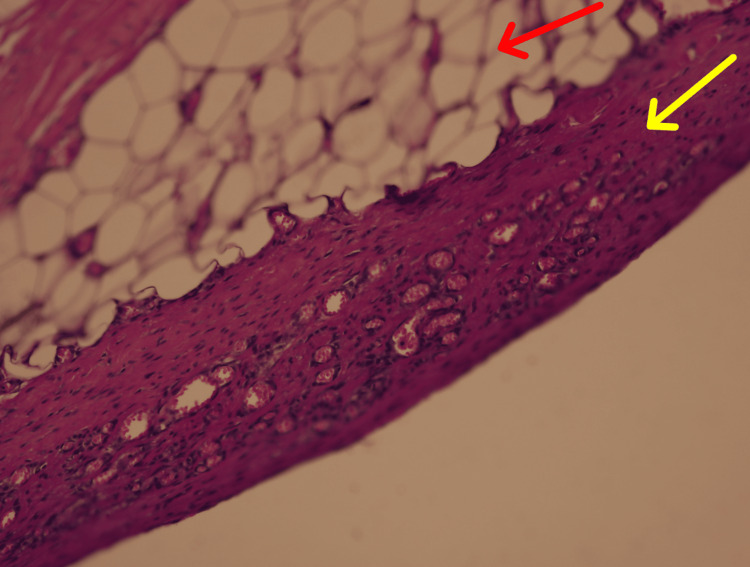
Fibrotic capsule (yellow arrow) and adipose tissue (red arrow) within the trochanteric abscess (20×, Sample 19-22-A1).

**Figure 5:**
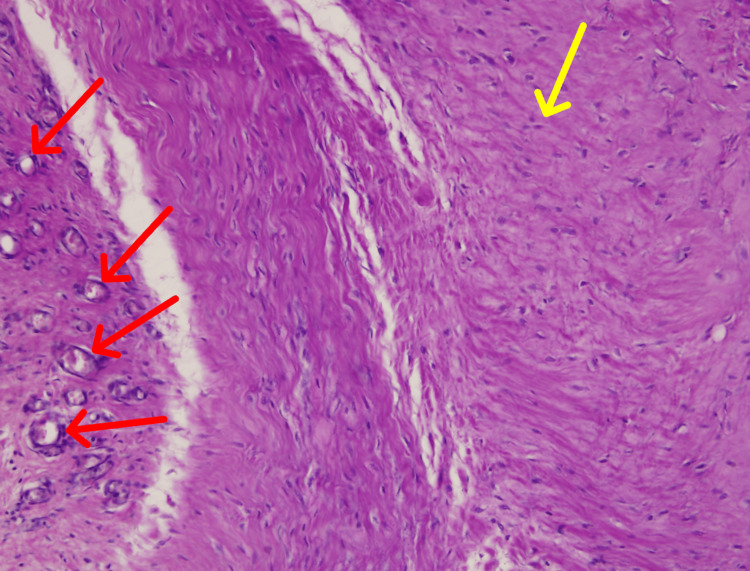
Dense irregular connective tissue stroma (yellow arrow) and arterioles (red arrows) within the trochanteric bursa (20×, Sample 19-42-A1).

These inclusions were surrounded by fibrotic tissue and occasional inflammatory cells, though no acute neutrophilic infiltrate, necrosis, or purulent exudate was identified. A well-formed fibrotic capsule was noted in Sample 19-22-A1 (Figure 4), and areas of irregular connective tissue with degenerative change were present in Sample 19-42-A1 (Figure 5).

Overall, the histological features were consistent with chronic inflammation and fibrosis rather than acute infection or malignancy. The absence of cartilage or bone formation argued against synovial chondromatosis, though the presence of inclusion bodies may represent proteinaceous degeneration secondary to chronic bursitis.

## Discussion

The results of the histological examination do not provide a definitive diagnosis of the clinical condition associated with the abscess. The inconclusive histological examination and the lack of a full medical background of the subject have made it impossible to identify the cause of the cadaveric anomaly. Our histopathological examination, gross findings, and literature search support the diagnosis of a chronic inflammatory process within the trochanteric bursa, possibly representing the sequelae of repetitive microtrauma or ischemic degeneration. Table 1 summarizes our findings.

**Table 1:** Summary of clinical, gross, and histopathological findings.

Characteristics	Findings	Clinical significance
Anatomic location	Left extra-acetabular peri-trochanteric region adjacent to the greater trochanter	Extra-articular process involving the trochanteric bursa rather than intra-articular hip joint pathology
Gross appearance	Fluid-filled cavity; irregular yellow-brown soft tissue fragments; multiple small nodules; loose bodies; black inclusions	Consistent with chronic bursal process; mimics abscess, mass, or degenerative synovial pathology
Overall histologic pattern	Chronic fibrosis, proteinaceous degeneration, vascular remodeling	Most consistent with chronic bursitis with possible secondary synovial process
Fibrosis	Dense fibrotic connective stroma; well-formed fibrotic capsule (Sample 19-22-A1)	Suggests a chronic inflammatory process rather than an acute infection
Adipose tissue	Abundant adipose tissue interspersed within the fibrotic stroma	Typical peri-trochanteric soft tissue composition
Vascularity	Dense vascularization with small-caliber vessels	Supports chronic inflammation and tissue remodeling
Inflammatory cells	Occasional inflammatory cells; absence of acute neutrophilic infiltrate	No evidence of acute bacterial abscess
Inclusion bodies	Numerous darkly pigmented, round-to-oval proteinaceous inclusions (highest density in Sample 19-45)	May represent degenerative proteinaceous material or rice-body–like formations associated with chronic synovitis
Necrosis	None identified	Argues against active infection
Malignancy	No evidence of malignancy	Excludes neoplastic etiology
Cartilage/Bone formation	Absent	Does not support primary synovial chondromatosis

Trochanteric bursitis demonstrates a higher prevalence in women, affecting 15% unilaterally and 8.5% bilaterally, whereas corresponding rates in men are 6.6% and 1.9% [6]. Due to limited reporting, the true prevalence of extra-acetabular trochanteric abscess has not been established. Potential etiologies leading to the subject’s condition include (1) chronic trochanteric bursitis secondary to mechanical stress or prolonged immobility, (2) synovitis or inflammation of the synovial membrane of the bursa, leading to (3) degenerative changes such as secondary synovial osteochondromatosis.

Supporting the first etiology, numerous studies have confirmed a significant correlation between middle-aged or elderly females and trochanteric bursitis [7]. Wide pelvis or hormonal effects among females may contribute to an increased susceptibility to developing chronic trochanteric bursitis [8]. Increased quadricep angle between the line of pull of the quadriceps and patellar tendon creates a greater lateral vector force on the femur, increasing tension over the greater trochanter [8]. Repeated friction or compression of the IT band and gluteal tendons against the greater trochanter combined with relative hip adduction during gait and gluteal tendon overload may contribute to this chronic etiology [2,8-11]. Additionally, estrogen deficiency among older women can lead to reduced collagen synthesis, increased catabolic signaling, altered matrix remodeling, and susceptibility to tendon degeneration, increasing vulnerability to overload or injury [12]. With trochanteric bursitis being more prevalent in developed countries and systemic inflammation being correlated with sedentary behavior, possible immobility of this patient may have caused chronic bursitis [9,13]. Fascia in this greater trochanteric region is susceptible to bursitis following repetitive pressure, such as due to poor positioning of the hips in immobile patients.

Synovitis or inflammation of the bursa or joint lining can accompany bursitis and may be the source of inclusion bodies [14]. Synovitis may be caused by overuse, trauma, or conditions such as rheumatoid arthritis and osteoarthritis. Inclusion bodies are generally related to synovial inflammatory conditions such as rheumatoid or seronegative inflammatory disorders [15]. Proposed mechanisms of pathogenesis include sloughing from the microinfarcted synovium, de novo formation in synovial fluid, and entrapment with collagen formation [16-18].

These combined findings are in accordance with our hypothesis that the prevalence of a rare anatomic bursitis may be explained by a deeper condition or disease causality. A theory that would fit this presentation includes synovial osteochondromatosis, a rare but benign monoarticular disease that creates changes in the lining of a joint [19]. Synovial osteochondromatosis typically affects large joints of the body, including the knee, elbow, shoulder, and hip. Similar to the intra-articular synovial chondromatosis described by Efrima et al., who demonstrated loose bodies and synovial proliferation within the hip joint, our findings support the concept that chronic synovial-lined structures in the peri-trochanteric region may undergo degenerative metaplastic change, although in our case, the process appears extra-articular and without definitive cartilaginous ossification [19]. Loose bodies or nodules are thought to form as part of mechanical changes and the degenerative processes of these joints. Huang et al. described “Fried Rice” patterned inclusion bodies inside the trochanteric bursa along with chronic synovial degeneration without evidence of tuberculosis, infection, or rheumatoid arthritis to support the diagnosis of idiopathic degenerative secondary process affecting the trochanteric bursa, similar to our study [15]. Several, small, free-floating cartilaginous nodules are characteristic of synovial osteochondromatosis, as well as metaplasia of the cartilage within the joint, and the large effusion that was present at the time of the gross dissection. Most similar to our presentation is the secondary synovial osteochondromatosis described by Peh et al., in which mechanical irritation and shedding of cartilaginous fragments induced synovial chondrometaplasia within a reactive bursa [20]. This further supports the possibility that chronic trochanteric bursitis may incite a secondary synovial metaplastic process in the peri-trochanteric region as described in our case study. Although the cause of synovial osteochondromatosis is unknown, secondary synovial osteochondromatosis is known to occur in older individuals.

## Conclusions

The findings in this case describe a rare extra-acetabular trochanteric bursa abscess identified during cadaveric dissection, characterized by chronic fibrotic change, proteinaceous inclusion bodies, and absence of acute infection or malignancy. The gross histopathological findings are most consistent with a chronic inflammatory bursal process, likely resulting from prolonged mechanical stress, synovial inflammation, or degenerative change. Presence of inclusion bodies and loose nodular material raises the possibility of a secondary synovial osteochrondromatosis as an underlying or contributory process. This report highlights the diagnostic complexity of extra-articular hip pathology and presents the importance of considering chronic bursitis and degenerative synovial conditions in peri-trochanteric lesions.
